# Isolated Posterior Medial Ankle Dislocation with Associated Os Trigonum Dislocation after Low-Energy Mechanism

**DOI:** 10.1155/2020/5026058

**Published:** 2020-01-27

**Authors:** Devan Irving, Brent Geers, Bruce Lawrence

**Affiliations:** Ascension Genesys Hospital, 1 Genesys Parkway, Grand Blanc, MI. 48439, USA

## Abstract

We describe a case of an isolated posteromedial ankle dislocation, without malleolar fracture, with associated dislocation of an os trigonal process after a low-energy tennis injury. We demonstrate that nonoperative treatment results in excellent functional outcome scores with minimal arthritic progression at 2 years of follow-up. We discuss pathoanatomic risk factors of pure dislocations and propose that an os trigonum is a risk factor for isolated dislocations of the ankle.

## 1. Introduction

Ankle dislocation without an associated fracture (pure ankle dislocation) is a rare injury accounting for only 0.065% of all ankle injuries [1]. Due to its rarity, pure ankle dislocations are not well understood with only case reports and small case series being described in the literature [2–10]. Even more unusual is a pure ankle dislocation with associated dislocation of an os trigonal process. This phenomenon has been described only once by Segal et al., in conjuncture with an anterior dislocation of the ankle [11]. Currently, risk factors for pure ankle dislocation are thought to be ligamentous hyperlaxity, medial malleolar hypoplasia, talar undercoverage, and history of prior ankle sprains [4, 6–9]. We present a case of posterior ankle dislocation with associated os trigonal dislocation following low-energy trauma and discussion of an os trigonal process as a possible risk factor for pure ankle dislocation.

## 2. Case Report

A 17-year-old female, LL, presented to our level 2 emergency room with pain and gross deformity of her left ankle. She sustained a plantarflexion and inversion injury while playing tennis. She reported pain in the ankle but denied paresthesia or loss of strength distal to the injury. She did not have any prior medical or surgical history and denied allergies to medications. She had met all developmental milestones at appropriate times. She and her parents denied any family history of hypermobility syndromes. She had no history of pain, instability, recurrent sprains, or prior functional issues in the affected ankle.

**Figure 1:**
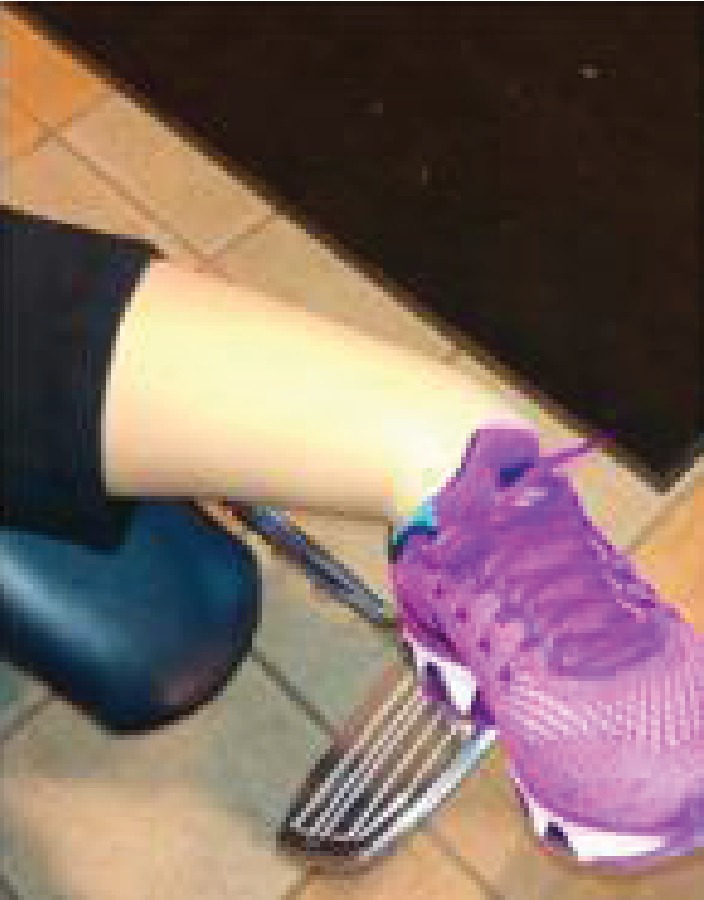
Gross internal rotation and inversion injury of the left ankle.

**Figure 2:**
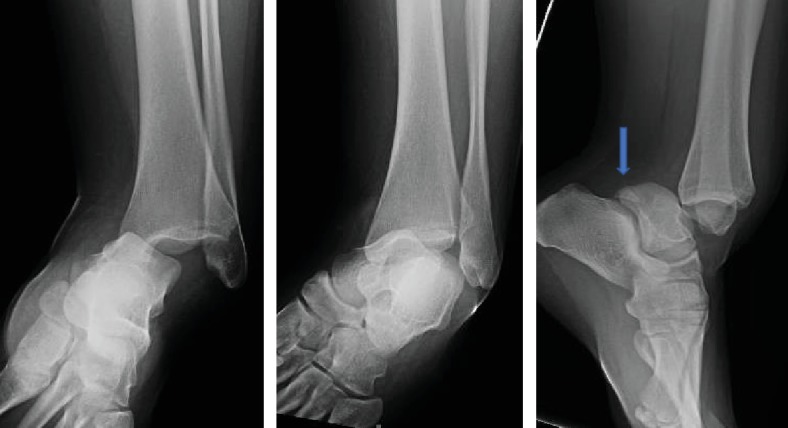
AP, lateral, and mortise radiograph demonstrating posteromedial ankle dislocation. The arrow on lateral view points towards double density over posterior talus representing displaced os trigonum.

**Figure 3:**
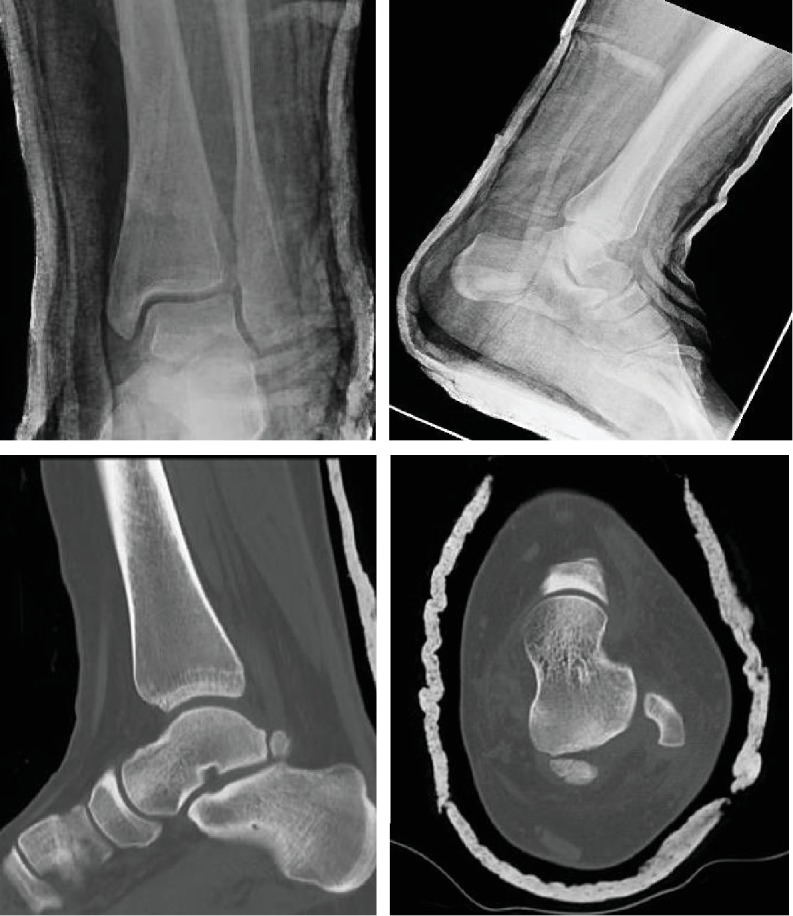
Mortise and lateral radiographs demonstrating reduced tibiotalar joint. Axial and lateral CT scans demonstrate displaced os trigonum without the fracture.

Physical examination demonstrated an inversion and internal rotation deformity of the ankle (Figure 1). The lateral malleolus was prominent with a 1 cm by 0.5 cm overlying superficial abrasion, which did not extend deeper than the epidermis. Distally, her foot was neurovascularly intact with +2/4 pulses in the dorsalis pedis and posterior tibial arteries with brisk capillary refill and sensation present in all nerve distributions. Radiographs of the left ankle were obtained and demonstrated a posteromedial ankle dislocation without malleolar fracture (Figure 2). On the lateral view, a double density over the posterior process of the talus was noticed and suspected to be an anteriorly displaced os trigonum. The patient was sedated with IV ketamine, and closed reduction of the ankle was performed. Reduction maneuver consisted of flexion of the knee to 90 degrees, anterior translation, and external rotation of the foot with posteriorly directed counter pressure on the tibia. The ankle was not stressed after reduction. A well-padded, short-leg posterior splint with stirrups was placed with the ankle in a neutral dorsiflexed position. Post reduction radiographs demonstrated a congruent tibiotalar joint reduction and reduced position of the os trigonum. However, persistent lucency of 2 mm between the os trigonum and the talar body remained. Post reduction computed topography was obtained and demonstrated no fracture, but well corticated and unfused posterior lateral process of the talus, consistent with an os trigonum (Figure 3).

The patient was treated nonoperatively with nonweight-bearing management initially in a short leg splint for two weeks and then cast for 4 weeks. At 6 weeks, she began to weight bear as tolerated in a walking boot. She resumed all normal activities by 3 months.

**Figure 4:**
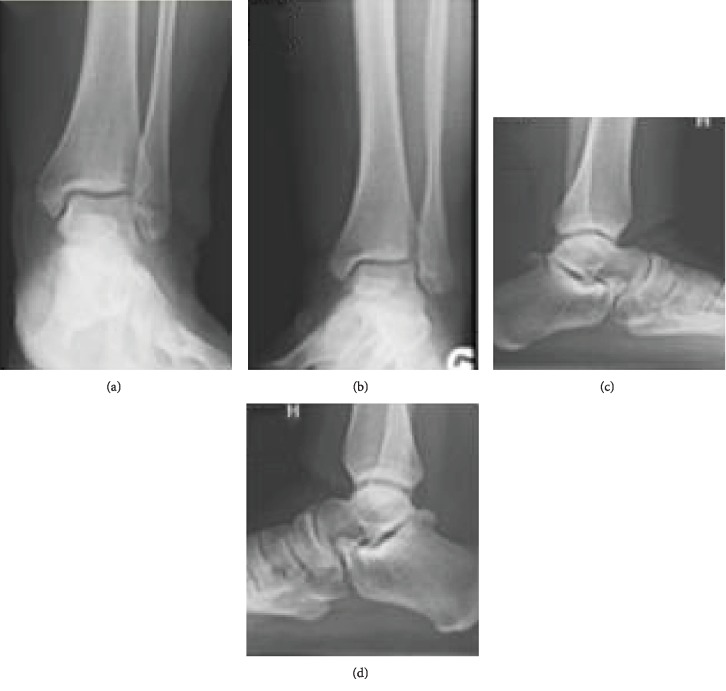
(a) AP view of the left ankle demonstrating mild medial tibial and talar osteophyte formation. (b) Mortise view of the left ankle again demonstrating medial osteophyte formation. (c) Lateral view of the left ankle demonstrating anterior tibial osteophyte formation and mild narrowing with mildly displaced os trigonum. (d) Lateral view of right ankle demonstrating nondisplaced os trigonum.

The final follow-up was 2 years from initial injury at which time the patient was very happy with her outcome. She admitted to mild, painless swelling in the injured ankle after activity and the occasional sensation of painless instability approximately once every 2 months. She was able to return to playing tennis the season after her injury. The patient was assessed with The American Orthopedic Foot and Ankle Society (AOFAS) Ankle-Hindfoot Score, visual analog scale (VAS), and Beighton score for hypermobility [12–14]. She scored perfectly in all categories of the AOFAS (pain, function, and alignment) for a final score of 100/100 points, indicating normal ankle function. She reported a 0/10 VAS score. Finally, the patient scored a 3 on the Beighton scale indicating no hypermobility. She had no pain over her os trigonum and no pain with forced plantar flexion. There was no laxity with drawer or talar tilt testing. Radiographs of bilateral ankles were obtained, and the injury side demonstrated medial talar and tibial osteophyte formation with mild anterior joint space narrowing. This is consistent with Kellgren-Lawrence Grade 1 changes of post traumatic osteoarthritis [15]. Medial malar hypoplasia was not present using the method described by Elise et al. [16]. Talar coverage was also found to be within normal limits according to the method described by Shaik et al. [7]. Notably, radiographs of the contralateral ankle also showed an os trigonum. When comparing os trigonums, the injured left side demonstrated more diastasis between the talus and os trigonum than the uninjured right side (Figure 4).

## 3. Discussion

Pure ankle dislocations and bilateral os trigonums are uncommon entities. Pure ankle dislocations represent only 13 per 20,000 ankle injuries, with the two most common causes being sporting injuries and motor vehicle accidents [1]. The rarity of this injury is attributed to the mechanical stability of the ankle mortise, with the ligamentous structures being relatively stronger than the boney malleoli. This results in the fracture of the malleoli prior to disruption of the ligaments and consequently a fracture dislocation rather than a pure dislocation [2]. Pure dislocations are classified by the direction of talar displacement: anterior, posterior, medial, or lateral. Posteromedial displacement is the most common, accounting for 46% [1]. In posteromedial dislocations, the deltoid ligament generally remains intact leading to the more medial orientation of the talus. Generally, this allows for avoidance of talar avascular necrosis because of the maintained talar blood flow through the deltoid ligament [1]. In our patient, we suspect the deltoid ligament remained intact given the posteromedial displacement and the lack of talar collapse at 2 years of follow-up. The exact mechanistic sequence of a posterior pure dislocation is debated in the literature. There is consensus that the position of the foot during injury is inversion and extreme plantarflexion. However, there is disagreement on how the talus comes to reside posteriorly. Fernandez, in his cadaveric study, was able to demonstrate pure dislocation only through anterior extrusion. Because of this, he reasoned that after anterior dislocation, the Achilles pulled the talus posteriorly [17]. Conversely, other authors feel that in the unstable equinus position, an appropriately directed force could result in direct posterior extrusion [7, 18–20].

An os trigonum is an accessory ossicle which overlies the posterior-lateral process of the talus. In normal development, the secondary talar ossification center (os trigonum) appears at 7-14 years and fuses to the talar body within 1 year of ossification [21, 22]. If fusion does not occur, an os trigonum results and is attached to the talus by a fibrous synchondrosis. Like pure ankle dislocations, bilateral os trigonum is uncommon. Zwiers et al. found the incidence of bilateral os trigonums to be only 14.3% in their retrospective CT evaluation of 628 patients [20]. Injury of the os trigonum can occur by two different mechanisms. Extreme equinus results in compression of the os between the calcaneus and posterior tibia, which may result in a “nutcracker” type of fracture. Conversely, an inversion injury can lead to avulsion of the os by tensioning through the posterior talofibular ligament (PTFL) [23].

We present a case of a pure posteromedial ankle dislocation with a dislocated os trigonum in the setting of low-energy trauma. To the author's knowledge, this is the first case of this specific injury to be presented in the literature. Segal and colleges described a nearly opposite case of a pure anterior ankle dislocation with os trigonum dislocation following a motor vehicle accident. Segal hypothesized that in his case, violent plantarflexion with the calcaneus resting on the motorcycles' foot peg resulted in anterior dislocation and a compression-type fracture of the os trigonum [11]. In lower energy trauma, anatomic variations and prior injuries are thought to predispose individuals to sustaining this rare injury. Current risk factors include medial malleolar hypoplasia, talar undercoverage, hypermobility, and history of recurrent ankle sprains [6–9, 21].

Our patient demonstrated no known risk factors, and we believe the presence of an os trigonum may be a new risk factor. Normally, the PTFL short fibers run from the fibula to the lateral talus whereas the long fibers run from the fibula to the posterior-lateral process of the talus [24]. If an os trigonum is present, then the long fibers attach directly onto the os, which is only attached to the talus by a synchondrosis [25]. We hypothesize that this results in an anatomic “weak link” in the ankle due to the synchondrosis being less robust than the bony malleolus and the normal PTFL attachment. The position of equinus and inversion is known to cause both injuries to the relatively weak ATFL, as well as, avulsion of the os trigonum through its synchondrosis [23, 26]. In this scenario, the lateral ligamentous complex would be significantly weakened, possibly pre-disposing to posterior dislocation [27]. This theory is supported by Gursoy et al.'s findings in their 2015 retrospective MRI study. In this study, they found an association between the presence of an os trigonum and an increase in signal intensity throughout the lateral ankle ligaments, which pointed towards chronic instability [25]. Despite this, pure ankle dislocation has not been reported as a complication following excision of symptomatic os trigonum without repair of the PTFL insertion. However, the literature on the subject is primarily small level IV studies [28–34]. Given that pure ankle dislocation is such a rare entity, it is possible these small studies were underpowered to detect its increased rate postoperatively.

Furthermore, our study corroborates the findings of multiple other authors in that ankle dislocations, without malleolar fracture, can be treated nonoperatively with good results [2–10]. This is likely due to the intact deltoid ligament and maintained blood supply. At two years, our patient reported a perfect score on the AOFAS and 0/10 pain on VAS. Radiographically, her ankle demonstrated only very mild arthritic changes which were likely a result of the initial injury. She occasionally experienced sensations of instability but this could not be reproduced on physical exam and was not symptomatic enough to prevent her from returning to sport. The injured ankle did continue to demonstrate slight widening of the os trigonum synchondrosis compared to the contralateral side, but this was asymptomatic.

In conclusion, pure ankle dislocation with associated os trigonum dislocation is a rare entity. The pathologic mechanism of pure ankle dislocations is not well understood. Known risk factors for pure dislocation include hyperlaxity, medial malleolar hypoplasia, talar undercoverage, and history of recurrent ankle sprains. We propose that the presence of an os trigonum may be a risk factor for pure ankle dislocation due to alternations in the attachment of PTFL.
